# Chemical Profile and Antimicrobial Activity of the Fungus-Growing Termite Strain *Macrotermes Bellicosus* Used in Traditional Medicine in the Republic of Benin

**DOI:** 10.3390/molecules25215015

**Published:** 2020-10-29

**Authors:** Dima Hammoud Mahdi, Jane Hubert, Jean-Hugues Renault, Agathe Martinez, Andreas Schubert, Kathrin Monika Engel, Blaise Koudogbo, Zacharie Vissiennon, Virgile Ahyi, Karen Nieber, Cica Vissiennon

**Affiliations:** 1 Inter-Regional University of Industrial Engineering Biotechnologies and Applied Sciences, IRGIB Africa, Cotonou 07 BP 231, Benin; blaise.koudogbo@gmail.com (B.K.); vissiennon@gmx.de (Z.V.); ahyivirgile@yahoo.fr (V.A.); 2 Medical Faculty, Institute of Medical Physics and Biophysics, Leipzig University, Härtelstr 16-18, 04107 Leipzig, Germany; kathrin.engel@medizin.uni-leipzig.de; 3 CNRS ICMR UMR 7312, Université de Reims Champagne Ardenne, BP 1039, 51687 Reims CEDEX 2, France; jane.hubert@univ-reims.fr (J.H.); jh.renault@univ-reims.fr (J.-H.R.); agathe.martinez@univ-reims.fr (A.M.); 4 NatExplore SAS, 51140 Prouilly, France; 5 Fraunhofer IZI, Institute for Cell Therapy and Immunology, Perlickstraße 1, 04103 Leipzig, Germany; andreas.schubert@izi.fraunhofer.de; 6 Medical Faculty, Institute of Pharmacy, Leipzig University, Brüderstr. 34, 04103 Leipzig, Germany; nieberkaren@gmx.de

**Keywords:** termite, *Macrotermes bellicosus*, traditional medicine, antibacterial activity, hydroquinone, methylhydroquinone, Republic of Benin

## Abstract

The fungus growing termite species *Macrotermes bellicosus* (*M. bellicosus*) is used in nutrition and traditional medicine in the Republic of Benin for the treatment of infectious and inflammatory diseases. Previous findings demonstrated evidence of anti-inflammatory and spasmolytic properties of *M. bellicosus*. The aim of the present study was to evaluate the antimicrobial potential of different extracts of *M. bellicosus* samples and determine the chemical profile of an ethanolic *M. bellicosus* extract. Chemical profiling was conducted using centrifugal partition chromatography and ^13^C-NMR, followed by MALDI-TOF MS. Major identified compounds include hydroquinone (HQ), methylhydroquinone (MHQ), 3,4-dihydroxyphenethyl glycol (DHPG), *N*-acetyldopamine (NADA) and niacinamide. The fatty acid mixture of the extract was mainly composed of linoleic and oleic acid and highlights the nutritional purpose of *M. bellicosus*. Using the Kirby–Bauer disc diffusion and broth microdilution assay, an antibacterial activity of *M. bellicosus* samples was observed against various clinical strains with a highest growth inhibition of *S. aureus*. In addition, HQ and MHQ as well as fractions containing DHPG, niacinamide and NADA inhibited *S. aureus* growth. The reported antimicrobial activity of *M. bellicosus* and identified active substances provide a rationale for the traditional medicinal use of *M. bellicosus*.

## 1. Introduction

Though insects have been widely used throughout history for medical treatment on nearly every continent, relatively little ethnopharmacological research has been conducted in comparison to medicinal plants [1]. One example are fungus-growing termites and derived products (termite mound and fungus comb) which are considered essential in tropical Africa because of their uses in nutrition and traditional medicine of various societies [2]. *Macrotermes bellicosus (Smeathman*, *1781)*, a species belonging to the *Macrotermitidae* is native to the Republic of Benin where it is considered a natural resource for nutritional, cultural and medicinal purposes. The main consumed castes are winged, soldier and occasionally queen termites for cultural and rituals events [3,4]. In a previous ethnomedicinal survey, it was found that *M. bellicosus* is mainly used to treat digestive disorders, mumps, snake bites, cough, diarrhea, dysentery and pulmonary infection in different districts of northern Benin [4]. Most of the reported traditional indications include diseases with infectious and thus inflammatory etiology and, depending on the treated disease, the termites are applied orally or topically as dried and powdered preparations. The anti-inflammatory effects of *M. bellicosus* were investigated and demonstrated in a prior study [4]. However, the pharmacological evaluation of *M. bellicosus* with respect to antimicrobial activity and the determination of active compounds that could contribute to the bioactivity remains unexplored. Termites are a group of social insects that are classified at the order of Isoptera. They live in large colonies and are hierarchically organized according to a system of well-defined functional castes with a differentiated morphology: workers, soldiers, winged reproducers, queen and king [5]. As with other eusocial species, it is known that they apply different mechanisms of disease control for their adaptation to the infection risks from pathogenic bacteria, fungi, and other microbes that thrive in their nest environment [6]. These mechanisms include mutual grooming, removal of diseased individuals from the nest, the innate and adaptive immune responses of colony members and antibiotic glandular secretions [6]. The latter highlights the interest for chemical investigation of *M. bellicosus* with the aim to identify bioactive compounds with antimicrobial properties.

In view of the above consideration, the present study aimed to investigate the antimicrobial potential of an *M. bellicosus* ethanolic extract in a first instance. Then, the chemical profile was determined in order to identify potential bioactive as well as nutritive compounds. Lastly, some of the biologically active metabolites, that could contribute to the antimicrobial activity, were identified. Chemical profiling was performed using centrifugal partition chromatography and ^13^C Nuclear Magnetic Resonance (^13^C-NMR) followed by matrix-assisted laser desorption/ionization time-of-flight mass spectrometry (MALDI-TOF MS) to evaluate the lipid composition. The antimicrobial properties of *M. bellicosus* extracts as well as fractions thereof and identified compounds were screened against selected pathogenic microorganisms using the Kirby–Bauer disc diffusion method and further characterized with the broth microdilution assay.

## 2. Results and Discussion

### 2.1. Chemical Profile

The crude ethanolic extract of *M. bellicosus* (soldier caste) collected from Abomey-Calavi (MBE_AB_) was submitted to a ^13^C-NMR dereplication workflow in order to identify its major metabolites [7]. First, 1.4 g MBE_AB_ was fractionated by centrifugal partition chromatography (CPC) using the ternary two-phase solvent system ethyl acetate/acetonitrile/water (3:3:4, *v*/*v*/*v*) in ascending mode, and a total of 12 fractions was obtained (Table 1).

**Figure 1:**
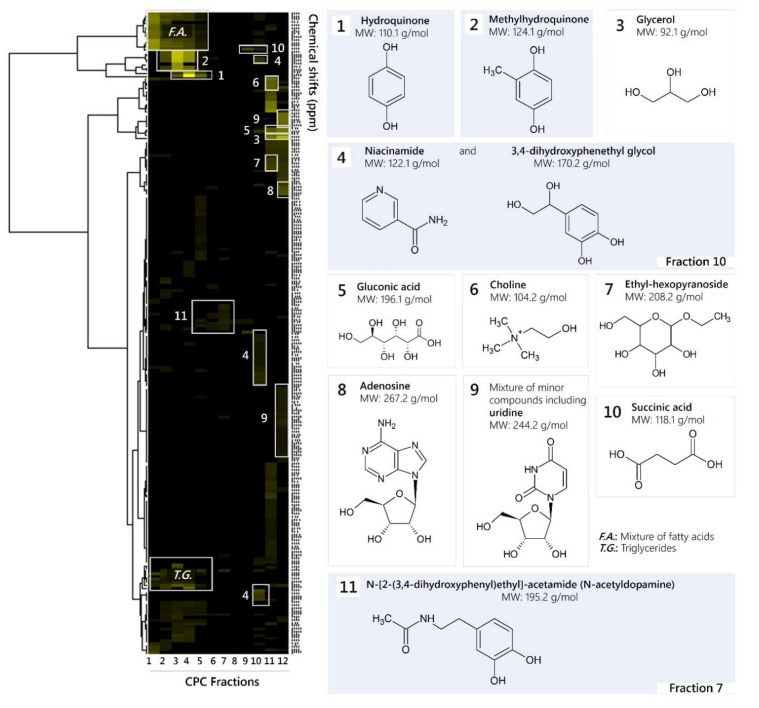
^13^C-NMR chemical shift clusters obtained by applying hierarchical clustering analysis on CPC fractions of an ethanolic *M. bellicosus* extract (soldier caste) collected from Abomey-Calavi and identified chemical structures. Fractions or pure substances that were assayed for antimicrobial activity are highlighted in blue.

The mass of each recovered fraction (4.8–892.4 mg) was enough to record ^13^C-NMR spectra with good resolution in about 20 min. The spectra were then submitted to an automatic peak picking and therefore reduced to a list of peak coordinates. The peak intensity values were stored in 0.2 ppm wide, initially empty, chemical shift intervals called bins, so that each one corresponded to a chemical shift value. The content of the bins was stored in a table: bin index in rows, fraction index in columns, peak intensities for values in table. In the present work, this-NMR data treatment resulted in a table with 12 columns (one per fraction) and 217 rows (one per chemical shift bin containing at least one ^13^C-NMR signal in at least one fraction). This table was submitted to HCA on the rows. In this way, statistical correlations between ^13^C-NMR resonances belonging to a single structure within the fraction series were visualized as chemical shift clusters beneath the corresponding dendrogram (Figure 1). Each cluster was finally assigned to a molecular structure with the help of a calculated ^13^C-NMR chemical shift database of natural metabolites and all database proposals were validated (or rejected) through simultaneous interpretation of 2D-NMR spectra (HSQC, HMBC and COSY). As a result, 13 major compounds were identified, including hydroquinone (HQ), methylhydroquinone (MHQ), 3,4-dihydroxyphenethylglycol (DHPG), *N*-acetyldopamine (NADA, *N*-[2-(3,4-dihydroxyphenyl)ethyl]acetamide), niacinamide, ethyl-hexopyranoside, adenosine, succinic acid, gluconic acid, choline, glycerol, oleic acid and linoleic acid. NMR data are presented in the Supplementary Material.

The results confirmed the findings of a preliminary GC-MS analysis performed in a previous study, which reports the presence of putative compounds, such as quinones, sugar derivatives, fatty acids, and steroid-like compounds [4]. Most of the identified compounds are reported for the first time in *M. bellicosus* as former studies performed on this termite species focused exclusively on its nutrient composition [8,9]. However, HQ was found in the labial gland of other termite species [10,11,12]. Hydroquinone and other hydroquinone derivatives, which are secreted by the labial gland, are known to serve as food-marking pheromones in lower and defensive agents in higher concentrations [13,14]. NADA is present in several insect species and acts as a precursor for quinonoid-sclerotizing agents in insect cuticle [15,16].

**Figure 2:**
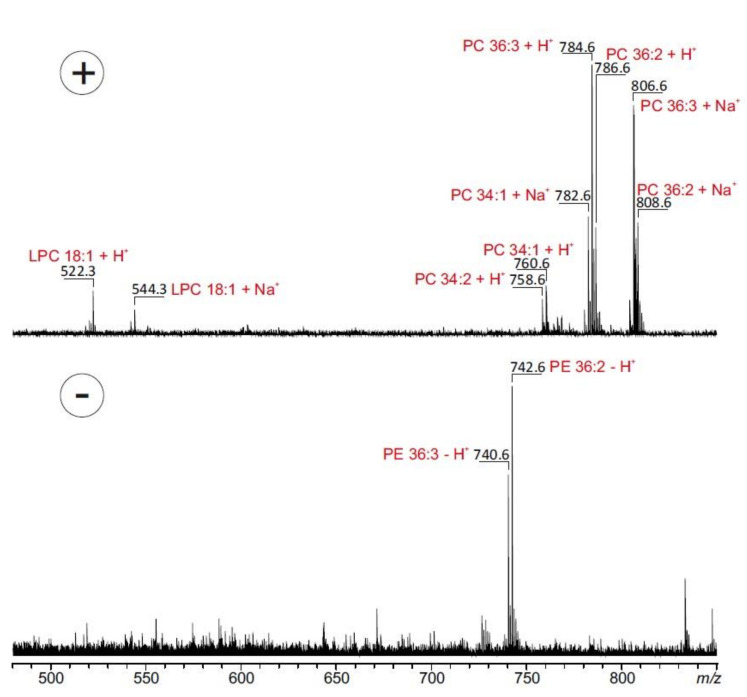
MALDI-TOF mass spectra of ethanolic *M. bellicosus* extract (soldier caste) collected from Abomey-Calavi. Spectra were recorded in the positive (**upper** part) and negative ionization mode (**lower** part) in the presence of 2,5-dihydroxybenzoic acid (0.5 M) dissolved in methanol as matrix. The most prominent peaks are assigned to the underlying phospholipid species. Lyso-phosphatidylcholine (LPC), phosphatidylcholine (PC) and phosphatidylethanolamines (PE).

In a second set of experiments, MBE_AB_ was submitted to MALDI-TOF MS analysis in order to specify the fatty acid composition (Figure 2). Spectra of the organic extract were recorded in the positive and in the negative ion modes. Spectra obtained in the positive ion mode showed phosphatidylcholine (PC) moieties at *m*/*z* 758.6, 760.6, 782.6, 784.6, 786.6, 806.6 and 808.6 corresponding mainly to the proton and sodium adducts of PC 34:1 (16:0/18:1), PC 34:2 (16:0/18:2), PC 36:2 (18:1/18:1 or 18:0/18:2) and PC 36:3 (18:1/18:2). A high amount of oleic acid (18:1) in the phospholipids was also demonstrated by the appearance of the referring *lyso*-phosphatidylcholine (LPC 18:1) (Figure 2).

Phosphatidylethanolamines (PE) were detected in the negative ion mode at *m/z* 740.6, 742.6 corresponding to PE 36:2 (18:1/18:1 or 18:0/18:2) and PE 36:3 (18:1/18:2). Fatty acids, such as linoleic acid and stearic acid, are known to be components of the frontal gland of some termite species, responsible for the biosynthesis of sex pheromones [17]. In line with our results, studies performed on *M. bellicosus* from Nigeria and Togo showed a high content of fatty acids with the presence of oleic acid, palmitic acid (16:0) and linoleic acid (18:2), which suggests that the lipid composition of the studied species is not largely affected by geological and environmental factors [18,19]. These results confirmed the assertion of some authors that insects and termites in particular are good sources of dietary lipids [20,21]. The crude fat content of *M. bellicosus* alongside its other nutritional constituents, such as protein, calcium, iron, zinc, and vitamins A and E, rationalize the wide-spread use of termites as the most widely consumed insects in Africa [8].

### 2.2. Antimicrobial Activity of Ethanolic M. bellicosus Extracts, Fractions and Major Compounds

Using the Kirby–Bauer disc diffusion assay as a preliminary screening test and qualitative technique, the antimicrobial potential of all prepared extracts from *M. bellicosus* samples were investigated based on their traditional use in the Republic of Benin against inflammatory and infectious diseases [4]. From all tested *M. bellicosus* extracts (termites of soldier and worker caste as well as termite mound and fungus comb) collected in Abomey-Calavi, Sekou, Lokossa, only extracts of the soldier caste of *M. bellicosus* collected at the three different sites in the Republic of Benin showed antimicrobial activity against different bacterial strains using the Kirby–Bauer disc diffusion assay (Table 2).

**Figure 3:**
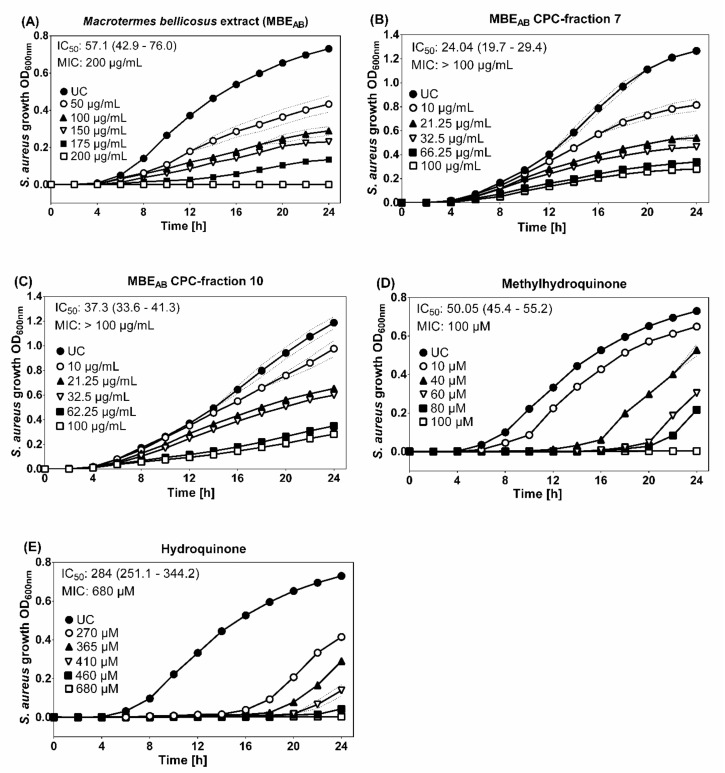
Growth curves of *S. aureus* incubated with ethanolic *M. bellicosus extracts* (soldier caste) collected in Abomey Calavi, MBE_AB_ (**A**); MBE_AB_ CPC-fraction 7 (**B**); MBE_AB_ CPC-fraction 10 (**C**) as well as the reference substances methylhydroquinone (**D**) and hydroquinone (**E**). The data shown are a compilation of four independent experiments done in triplicate with optical density at 600 nm (OD_600nm_). Data are presented as mean ± SD (dotted line); IC_50_ (95% confidence interval).

Variations in the antibacterial activity against microorganisms were observed depending on the collection site. In contrast to the ethanolic *M. bellicosus* soldier extract collected from Lokossa (MBE_LO_), which inhibited the activity of *K. pneumoniae*, the samples collected from Abomey Calavi (MBE_AB_) and Sekou (MBE_SE_) had no effect on the activity of this pathogen. Various factors could contribute to these variations and can be explained by differences in the metabolism and environmental conditions of the insects [22]. It is generally accepted that, unlike worker termites, soldiers are able to inhibit the proliferation of fungi in a longer term and their chemical defense arsenal is more variable than in any other insect taxon of comparable species number [23,24]. Soldier termites are equipped with a frontal gland, which represents their defense organ [25]. A variety of defensive molecules produced by this gland are known and mono-, sesqui- and di-terpenes, fatty acids, ketones, alcohols, aldehydes and aromatic compounds have been identified [25]. This is in accordance with the results obtained in this study which showed no antibacterial activity for the ethanolic extracts of worker termites but an inhibitory activity for the ethanolic extracts of soldier termites against various pathogenic microorganisms. However, no antifungal activity was observed against *Candida albicans*. The results obtained from the disc diffusion screening test identified MBE_AB_ as the most potent extract with a growth inhibition observed for *S. aureus* (IZD = 15 ± 0.2 mm). Thus, the broth microdilution assay was applied, which resulted in a MIC of 200 μg/mL MBE_AB_ for *S. aureus* growth (Figure 3).

Since HQ and MHQ were identified as the major components of MBE_AB_, the known antimicrobial activities of HQ and MHQ were tested in the same setting to determine their contribution to the antibacterial activity of the whole extract alongside with other potential ingredients. HQ (IC_50_ = 284 μM; MIC = 680 μM) and MHQ (IC_50_ = 50.05 μM; MIC = 100 μM) showed antibacterial activity against *S. aureus* with MIC comparable to results from previous studies [26,27]. Different antibacterial mechanisms, such as the destruction of the bacterial cell wall and membrane, resulting in increased permeability and intracellular substance leakage, as well as negative influence on gene expression and protein synthesis, could lead to the observed anti-*S. aureus* activity of HQ [28]. The stronger anti-*S. aureus* activity observed for MHQ indicates a structure-dependent activity. Further, antimicrobial properties have been discussed for analogues of acetamide and glycol, NADA, DHPG and niacinamide, whereby CPC-fraction 7 (containing NADA) and CPC-fraction 10 (containing DHPG and niacinamide) were assessed using the broth microdilution assay [29,30,31,32,33]. A moderate antibacterial activity could be demonstrated for the CPC-fraction 7 (IC_50_ = 24.04 μg/mL, MIC > 100 μM) and CPC-fraction 10 (IC_50_ = 37.28 μg/mL, MIC > 100 μM). DHPG is a naturally occurring endogenous intraneuronal metabolite of norepinephrine [34]. Its antimicrobial properties have been linked to the binding and inhibition of bacterial ATP synthase in a study that investigated the link between antimicrobial properties of phenolic compounds from *Olea europea* and bacterial ATP synthase [32]. Niacinamide (a form of vitamin B3), which was also identified in MBE_AB_ CPC-fraction 10 is known to have antimicrobial benefits on the skin as well. However, a recent study investigated its mechanism of action and discovered that niacinamide has no direct antimicrobial effect but boosts the activity of antimicrobial peptides (AMPs) [33]. The broth microdilution testing of MBE_AB_ CPC-fractions 7 and 10 led to the conclusion that NADA and DHPG, as major compounds of these fractions, could contribute to the antibacterial activity against *S. aureus*. However, the MIC were above 100 µg/mL and it must be noted that the determination of the susceptibility of pathogenic microorganisms to the test extracts has been hampered by the non-homogeneous solution of the extract in standard test media, partly due to the presence of lipids identified in its composition. Additional experiments on pure substances identified in the termite extract fractions are essential in order to get a better picture of their bioactivity. Antibacterial properties of the identified fatty acids, such as linoleic and oleic acid, may also slightly contribute to the observed effect in the *M. bellicosus* extract, as these substances at high concentration are known to inhibit the growth of *S. aureus* with a synergistic effect between the respective two fatty acids [35]. Choline-based ionic liquids, gluconic acid, succinic acid and uridine derivatives are also known for their antimicrobial properties. Therefore, choline, gluconic acid, succinic acid and uridine are other compounds identified in the *M. bellicosus* extract with potential antimicrobial contribution [36,37,38,39]. Referring to the literature and to the best of author’s knowledge, there is no documented relation between the other identified compounds and the anti-*S. aureus* activity.

The present findings contribute to the scientific knowledge on the traditional use of *M. bellicosus* in the treatment of *S. aureus*-associated diseases which is highly relevant in West Africa as a high proportion of *S. aureus* infections including wound infection, urinary tract infection, diarrhea and pneumonia have been reported in the Republic of Benin [40,41,42,43]. It was demonstrated that *S. aureus* is a causative agent of acute and diarrheal diseases in the Republic of Benin, which are often linked to a lack of hygienic practices in street food handling and the use of low quality water [42,43,44].

## 3. Materials and Methods

### 3.1. Chemicals and Reagents

Ethyl acetate (EtOAc) and acetonitrile (CH_3_CN) were purchased from Carlo Erba Reactifs SDS (Val de Reuil, France). Deionized water was used to prepare all aqueous solutions. Microbiological media were procured from Carl Roth^®^ GmbH & Co. KG (Karlsruhe, Germany) and phosphate buffered saline (PBS) pH 7.4 was obtained from Gibco, Thermo Fisher Scientific (Grand Island, NY, USA). All culture media were prepared and treated according to the manufacturer guidelines. Amoxicillin trihydrate and meropenem trihydrate (both from Sigma Aldrich, Taufkirchen, Germany) were used as reference antibiotics. All other chemicals used were of analytical grade and purchased from Sigma Aldrich.

### 3.2. Termite Materials

This study was performed on *M. bellicosus* soldier and worker termites as well as termite mound and fungus comb collected from different regions in the Republic of Benin during the month of February 2016 (Table 3). The samples were authenticated by Dr. Laura E. Y. LOKO, entomologist from the Faculté des Sciences et Techniques (FAST), Abomey Calavi University (UAC), Republic of Benin. Voucher specimens of the termite samples have been deposited at the Inter-Regional University of Industrial Engineering Biotechnologies and Applied Sciences, IRGIB, Cotonou, Benin with defined ID-numbers (AB-02-2016, SE-02-2016, LO-02-2016). A certificate for exportation was obtained from the “Ministry of Agriculture and Husbandry”, the Republic of Benin.

### 3.3. Preparation of Termite Extracts

Extracts of the *M. bellicosus* materials were prepared according to a method adapted from [45]. Briefly, 5 g of non-treated termite material or derived products (termite mound and fungus comb) were separately collected from the field and extraction was performed within 1 h after collection. The termite material as well as termite mound and fungus comb were stirred vigorously in 10 mL of ethanol (HPLC grade 90% *v*/*v*, Alfa Aesar, Ward Hill, MA, USA) and macerated for six days at room temperature. The different samples were subjected to sonication (15 min) followed by centrifugation (10 min, 6000 rcf) and the hydro-alcoholic phase was isolated from the residual deposit, filtered (Whatman^TM^ Grade 1 Qualitative filter paper pore size 11 µ) and the solvent was evaporated. As a result, extraction yield of around 200 µg was obtained for each extract. The dried extract was stored at −20 °C and dissolved in dimethyl sulfoxide (DMSO) for biological testing.

### 3.4. Chemical Profiling of M. bellicosus

#### 3.4.1. Centrifugal Partition Chromatography

A crude ethanolic extract of *M. bellicosus* (soldier caste) collected from Abomey-Calavi (MBE_AB_) was fractionated by centrifugal partition chromatography on a lab-scale FCPE300^®^ column of 303.5 mL capacity (Rousselet Robatel Kromaton, Annonay, France) containing 7 circular partition disks and engraved with a total of 231 oval partition twin cells (≈1 mL per twin cell). The liquid phases were pumped by a KNAUER Preparative 1800 V7115 pump (KNAUER Wissenschaftliche Geräte GmbH, Berlin, Germany). Fractions were collected by a Pharmacia Superfrac collector (Uppsala, Sweden). A two-phase solvent system composed of ethyl acetate/acetonitrile/water (3:3:4, *v*/*v*/*v*) was thoroughly equilibrated in a separatory funnel. The lower phase of the two-phase solvent system was used as the stationary phase (ascending mode) and the upper phase was used as the mobile phase. Flow rate was 20 mL/min and the column rotation speed was set at 1200 rpm. The extract (1.4 g) was solubilized in a 2:1 (*v*/*v*) mixture of the aqueous and organic phases of the two-phase solvent system. An isocratic elution of the mobile phase was performed in the ascending mode for 48 min. The column was finally extruded for 15 min. Fractions of 20 mL were collected over the whole experiment and combined according to their thin layer chromatography (TLC) profiles. As a result, 12 fractions were obtained (Table 1). TLC was performed on pre-coated silica gel 60 F254 Merck plates (Merck KGaA, Darmstadt, Germany) with the migration solvent system chloroform/ethylacetate/formic acid (50:30:2, *v*/*v*/*v*), visualized under UV light at 254 nm and 360 nm and by spraying the dried plates with 50% H_2_SO_4_ and vanillin followed by heating at 110 °C.

#### 3.4.2. ^13^C-NMR Analyses and Data Processing

Nuclear magnetic resonance (NMR) analysis and data interpretation was performed according to Hubert et al. (2014) [7]. An aliquot of each fraction from F1 to F12 (up to ≈20 mg) was dissolved in 600 µL DMSO-*d*_6_ and analyzed at 298 K on a Bruker Avance AVIII-600 spectrometer (Bruker BioSpin, Karlsruhe, Germany) equipped with a TXI cryoprobe. ^13^C-NMR spectra were manually phased and baseline corrected using the TOPSPIN 3.2 software (Bruker) and calibrated on the central resonance of DMSO-*d*_6_ (δ = 39.80 ppm). The absolute intensities of all ^13^C-NMR signals were automatically collected and binned across the spectra of the fraction series by using a locally developed computer script. The resulting table was imported into the PermutMatrix version 1.9.3 software (LIRMM, Montpellier, France) for hierarchical clustering analysis (HCA). The resulting ^13^C-NMR chemical shift clusters were visualized as dendrograms on a two-dimensional map (Figure 1). The higher the intensity of the ^13^C-NMR signals, the brighter the color on the map. For metabolite identification, each ^13^C-NMR chemical shift cluster obtained from HCA was manually submitted to the structure search engine of the database management software ACD/NMR Workbook Suite 2012 software, ACD/Labs, Ontario, Canada) comprising the structures and predicted chemical shifts of low molecular weight natural products (n ≈ 3000 in May 2018). Additional 2D-NMR experiments (HSQC, HMBC, and COSY) were performed on fractions containing putatively identified compounds to confirm the molecular structures proposed by the database at the end of the dereplication process.

#### 3.4.3. MALDI-TOF MS

The lipid composition of MBE_AB_ was investigated by matrix-assisted laser desorption/ionization time-of-flight mass spectrometry (MALDI-TOF MS). Lipids were extracted from the MBE_AB_ extract by adding 200 µL chloroform/methanol (1:1, *v*/*v*) according to the procedure of Bligh and Dyer (1959) [46]. After phase separation the lower (organic) phase was isolated using a Hamilton syringe. The organic extracts were directly used for MALDI-TOF analysis in order to avoid sample alterations. A solution of 2,5-dihydroxybenzoic acid (DHB, 0.5 M) in methanol was used as matrix [47]. Organic extracts were mixed 1:1 (*v*/*v*) with DHB and vortexed for good homogeneity. Subsequently, 0.75 µL of the respective solution were transferred onto an aluminum-coated MALDI target (Bruker Daltonics GmbH, Bremen, Germany). Instantly after air drying, positive and negative ion mass spectra were recorded on a Bruker Autoflex mass spectrometer (Bruker Daltonics) which utilizes a pulsed 50 Hz nitrogen laser emitting at 337 nm. The extraction voltage was 20 kV. Gated matrix suppression was applied to prevent saturation of the detector by matrix ions. For each mass spectrum 350 single laser shots were averaged. Laser-induced sample alterations were kept to a minimum by setting the laser energy only slightly above the threshold level. In order to enhance the resolution, all spectra were recorded in the reflector mode. Raw data were processed using the software “Flex Analysis” version 3.0 (Bruker Daltonics)

### 3.5. Antimicrobial Testing

#### 3.5.1. Microbiological Material

Different clinical strains; *Staphylococcus aureus* (*S. aureus*), *Escherichia coli* (*E. coli*), *Pseudomonas aeruginosa* (*P. aeruginosa*), *Klebsiella pneumoniae* (*K. pneumoniae*) and *Candida albicans* (*C. albicans*) were retrieved from the bacterial collection of the National University Hospital Centre (CNHU), Cotonou, in the Republic of Benin and used for the preliminary investigation of the antimicrobial activity using the Kirby–Bauer disc diffusion method. The test strain *S. aureus* (DSM 346) from the “German collection of microorganisms and cell culture” was used for the broth microdilution test.

#### 3.5.2. Kirby–Bauer Disc Diffusion Assay

In order to obtain a general picture of the antimicrobial potential of the extracts prepared from different *M. bellicosus* materials (termites of soldier and worker caste as well as termite mound and fungus comb) collected in Abomey-Calavi, Sekou, Lokossa (Table 3), the standardized Kirby–Bauer disc diffusion method was used as a preliminary screening test and qualitative technique prior to quantitative minimal inhibition concentration (MIC) determination using the broth microdilution method. The Kirby–Bauer disc diffusion assay was performed according to [48]. From the microbial strains in pure, fresh culture (18–24 h), inoculum was prepared in 1 mL sterile distilled water for each strain. The 0.5 McFarland solution (1–2 × 10^8^ CFU/mL) served as standard to prepare microbial inoculants. The microbial inoculum was uniformly spread using a sterile cotton swab on a sterile petri dish containing Mueller Hinton (HiMedia^®^, Mumbai, India) agar. Small filter paper discs of 6 mm in diameter were perforated and sterilized before impregnation with the different test extracts to obtain a final loading of 50 µg/disc and 100 µg/disc. Discs impregnated with 90% ethanol were used as solvent control. All discs were dried at 37 ± 2 °C for 2 h to completely remove the solvent and to prevent any effect of the ethanol. To perform the assay, the impregnated discs were gently and aseptically deposited (20 mm apart from each other) on the agar surface of the different microbial test strains. The susceptibility of the bacteria to the test compounds was determined by the formation of an inhibition zone after 18 h of incubation under aerobic conditions at 35 ± 2 °C. A commercially available amoxicillin disc 25 µg/disc (HiMedia^®^, Mumbai, India) was used as a reference. Inhibition zone diameters (IZD) of the microbial growth were measured in mm.

#### 3.5.3. Broth Microdilution Assay

Based on the results obtained with the Kirby–Bauer disc diffusion assay, the antimicrobial investigation was pursued with the quantitative broth microdilution assay. Since the highest growth inhibition was observed against *S. aureus* and *S. aureus*-associated diseases are highly relevant in the Republic of Benin, the test strain *S. aureus* (DSM 346) was selected [40,43]. The assay was performed in 96-well plates according to the guidelines of the Clinical and Laboratory Standards Institute, CLSI (M07-A10, 2015) [49]. Briefly, bacterial suspensions (5 × 10^5^ CFU/mL) were added to Mueller–Hinton Broth in the presence of different concentrations of the tested samples. Thus, stock solutions of the test samples were prepared in DMSO, of which appropriate dilutions were prepared in PBS with a final concentration of 0.5% DMSO. Growth curves of *S. aureus* were generated over 24 h by measuring the optical density OD_600_ on a UV-visible spectrophotometer every 15 min at 35 ± 2 °C and 425 cpm (Epoch 2, Biotek). The MIC was recorded as the lowest tested sample concentration that prevented the visible growth of the test strains after 24 h. Vehicle control (PBS + 0.5% DMSO) was considered and meropenem (5 µM) was applied as a reference control.

#### 3.5.4. Data Evaluation and Statistical Analysis

The antimicrobial experiments were carried out with four independent experiments performed in triplicate. Results are expressed as mean ± standard deviation. GraphPad Prism version 6.01 for Windows (GraphPad Software, San Diego, CA, USA) was employed for the statistical analysis. A concentration–response curve based on nonlinear regression yielded to the half maximal inhibitory concentrations (IC_50_) and 95% confidence intervals (95% CI). MIC data were analyzed using one-way analysis of variance (ANOVA) and Dunnett’s multiple comparisons test. A *p* value < 0.05 was considered significant.

## 4. Conclusions

In summary, the present study demonstrated that a *M. bellicosus* extract exerts antimicrobial activity against various clinical strains. Moreover, compounds identified for the first time in the termite species *M. bellicosus* include HQ, MHQ, NADA and DHPG, which contribute to the growth inhibition of *S. aureus*. The results indicated that the antibacterial activity of the identified bioactive compounds may provide a rationale for the traditional medical uses of *M. bellicosus*. This is the first pharmacological evidence on the antibacterial effects of *M. bellicosus.* While also taking into account its previously demonstrated anti-inflammatory effects, the combined data reinforce the relevance of the traditional medicinal use of this termite species [4]. In addition, the presence of fatty acids which are essential for human health highlights the nutritional relevance of the species.

## Figures and Tables

**Table 1:** Mass and global composition of the fractions produced by centrifugal partition chromatography (CPC) of an ethanolic *M. bellicosus* extract (soldier caste) collected from Abomey-Calavi. HQ—Hydroquinone, MHQ—Methylhydroquinone, NADA—*N*-acetyldopamine, DHPG—3,4-dihydroxyphenethyl glycol, Maj—Major; Med—Medium; Min—Minor. Column volume: 303.5 mL; Elution and extrusion flow rate: 20 mL/min; Rotation speed: 1200 rpm; Sample: 1.4 g of MBE_AB_.

CPC- Fractions Nº (Time in Minutes)	Mass (mg)	% Crude Extract	Composition
1 (8–9) elution	39.5	2.8	Mixture of fatty acids among which linoleic acid (Maj) + oleic acid (Maj) + triglycerides (Min)
2 (10) elution	24.0	1.7	MHQ (Maj) + mixture of fatty acids (Maj) + triglycerides + triglycerides (Med)
3 (11) elution	25.3	1.8	HQ (Med) + MHQ (Maj) + mixture of fatty acids (Med) + triglycerides (Med)
4 (12–13) elution	24.6	1.7	HQ (Maj) + MHQ (Med) + mixture of fatty acids (Min)
5 (14–15) elution	8.3	0.6	HQ (Maj) + mixture of fatty acids (Min)
6 (16–18) elution	7.4	0.5	HQ (Med) + NADA (Med)
7 (19–21) elution	7.5	0.5	NADA (Maj)
8 (22–25) elution	4.8	0.3	NADA (Maj)
9 (26–28) elution	5.6	0.4	Succinic acid (Maj)
10 (30–48) elution	13.7	1.0	DHPG (Maj) + niacinamide (Maj) + succinic acid (Maj)
11 (49–50) extrusion	892.4	63.7	Glycerol (Med) + gluconic acid (Med) + choline (Maj) + ethyl hexopyranoside (Med)
12 (52–fin) extrusion	112.5	8.0	Glycerol (Maj) + gluconic acid (Med) + adenosine (Med) + uridine (Min)

**Table 2:** Inhibitory zone diameters (IZD) of ethanolic *M. bellicosus extracts* (soldier caste*)* collected in Abomey-Calavi (MBE_AB_), Sekou (MBE_SE_) and Lokossa (MBE_LO_), Republic of Benin, compared to amoxicillin (AMX). Data are presented as mean ± SEM obtained from a compilation of four independent experiments done in triplicate. * *p* < 0.001 vs. untreated control. (-) means ‘‘no inhibition’’. S. aureus—Staphylococcus aureus, E. coli—Escherichia coli, P. aeruginosa—Pseudomonas aeruginosa, K. pneumoniae—Klebsiella pneumoniae, C. albicans—Candida albicans.

	Inhibitory Zone Diameters IZD (mm)						
	*Macrotermes bellicosus*						AMX
	50 µg/disc			100 µg/disc			25 µg/disc
	MBE_AB_	MBE_SE_	MBE_LO_	MBE_AB_	MBE_SE_	MBE_LO_	
*S. aureus*	10 ± 0.3 *	6 ± 0.2 *	6 ± 0.6 *	15 ± 0.2 *	12 ± 0.4 *	11 ± 0.2 *	15 ± 0.4 *
*E. coli*	6 ± 0.5 *	5 ± 0.4 *	4 ± 0.3 *	8.5 ± 0.6 *	8 ± 0.3 *	7 ± 0.6 *	13 ± 0.5 *
*P. aeruginosa*	7 ± 0.6 *	6 ± 0.3 *	6.5 ± 0.2 *	8 ± 0.3 *	9 ± 0.5 *	7 ± 0.3 *	12 ± 0.2 *
*K. pneumoniae*	(-)	(-)	4 ± 0.3 *	(-)	(-)	6 ± 0.4 *	12 ± 0.6 *
*C. albicans*	(-)	(-)	(-)	(-)	(-)	(-)	(-)

**Table 3:** Overview of collected *M. bellicosus* samples (including termite mound and fungus comb) and corresponding GPS coordinates.

Site of Collection	GPS Coordinates	Species
Abomey-Calavi	6°26′ N, 2°21′ E	*Macrotermes bellicosus*, *Smeathman* 1781
Sekou	6°37′ N, 2°13′ E	*Macrotermes bellicosus*, *Smeathman* 1781
Lokossa	6°38′ N, 1°43′ E	*Macrotermes bellicosus*, *Smeathman* 1781

## Supplementary Materials

The following are available online, Table S1: ^1^H and ^13^C-NMR chemical shifts of *gluconic acid*, Table S2: ^1^H and ^13^C-NMR chemical shifts of *choline*, Table S3: ^1^H and ^13^C-NMR chemical shifts of *glycerol*, Table S4: ^1^H and ^13^C-NMR chemical shifts of *ethyl-hexopyranoside*, Table S5: ^1^H and ^13^C-NMR chemical shifts of *adenosine*, Table S6: ^1^H and ^13^C-NMR chemical shifts of *hydroquinone*, Table S7: ^1^H and ^13^C-NMR chemical shifts of *methylhydroquinone*, Table S8: ^1^H and ^13^C-NMR chemical shifts of *3*,*4-dihydroxyphenethyl glycol*, Table S9: ^1^H and ^13^C-NMR chemical shifts of *N-[2-(3*,*4-dihydroxyphenyl)ethyl]-acetamide (N-acetyldopamine)*, Table S10: ^1^H and ^13^C-NMR chemical shifts of *niacinamide*, Table S11: ^1^H and ^13^C-NMR chemical shifts of *succinic acid*.
