# Supplementary material for: Chemical Profile and Antimicrobial Activity of the Fungus-Growing Termite Strain Macrotermes Bellicosus Used in Traditional Medicine in the Republic of Benin
Source: Molecules. 2020 Oct 29;25(21):5015. doi: 10.3390/molecules25215015 (PMC7662623; doi:10.3390/molecules25215015)
Supplement: Supplementary file 1 [file molecules-25-05015-s001.pdf]

Supplementary Material

NMR Data of identified compounds from 'an ethanolic *Macrotermes bellicosus* extract (soldier caste)' collected from Abomey-Calavi.

S1. ^{1}H and ^{13}C NMR chemical shifts of *gluconic acid*

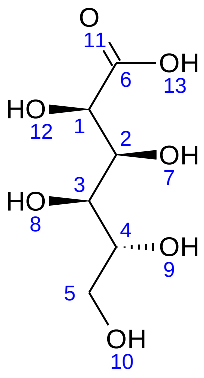

$C_6H_{12}O_7$	196.1 g/mol	DMSO-*d6*
Atom number	^{13}C (ppm)	^{1}H (ppm)
1	72.8	3.65
2	71.1	3.77
3	72.4	3.42
4	71.9	3.48
5	64.0	3.32/3.55
6	175.5	-

S2. ^{1}H and ^{13}C NMR chemical shifts of *choline*

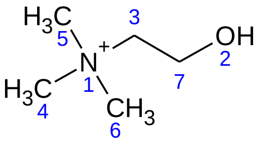

$C_5H_{14}NO$	104.2 g/mol	DMSO-d6
Atom number	^{13}C (ppm)	^{1}H (ppm)
3	67.3	3.40
4	53.5	3.11
5	53.5	3.11
6	53.5	3.11
7	55.6	3.83

S3. ^{1}H and ^{13}C NMR chemical shifts of *glycerol*

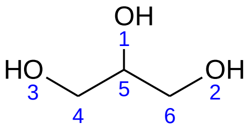

$C_3H_8O_3$	92.1 g/mol	DMSO-d6
Atom number	^{13}C (ppm)	^{1}H (ppm)
5	72.8	3.42
4/6	63.4	3.35/3.28

S4. ^{1}H and ^{13}C NMR chemical shifts of *ethyl-hexopyranoside*

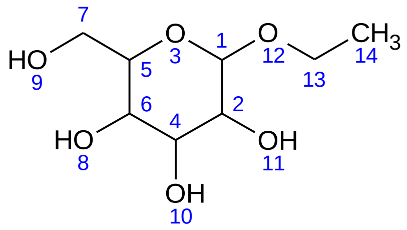

$C_8H_{16}O_6$	208.2 g/mol	DMSO-*d6*
Atom number	^{13}C (ppm)	^{1}H (ppm)
1	103.0	4.10
2	73.9	2.93
4	77.2	3.14
6	70.4	3.05
5	77.2	3.05
7	61.4	3.43/3.64
13	64.3	3.47/3.82
14	15.6	1.13

S5. ^{1}H and ^{13}C NMR chemical shifts of *adenosine*

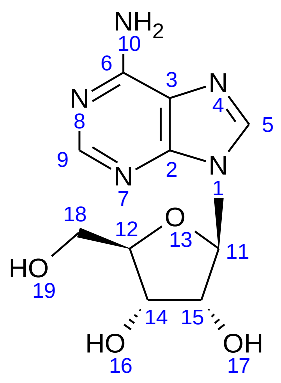

$C_{10}H_{13}N_5O_4$	267.2 g/mol	DMSO-*d6*
Atom number	^{13}C (ppm)	^{1}H (ppm)
2	149.5	-
3	119.7	-
5	140.4	8.36
6	156.6	-
9	152.9	8.13
11	88.3	5.87
15	74.0	4.59
14	71.1	4.15
12	86.4	3.96
18	62.1	3.55/3.65

S6. ^{1}H and ^{13}C NMR chemical shifts of *hydroquinone*

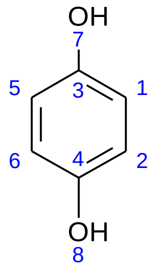

$C_6H_6O_2$	110.1 g/mol	DMSO-*d6*
Atom number	^{13}C (ppm)	^{1}H (ppm)
1/2/5/6	116.1	6.55
3/4	150.1	-

S7. ^{1}H and ^{13}C NMR chemical shifts of *methylhydroquinone*

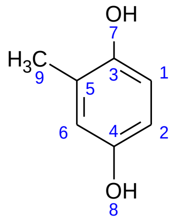

$C_7H_8O_2$	124.1 g/mol	DMSO-*d6*
Atom number	^{13}C (ppm)	^{1}H (ppm)
1	115.5	6.53
2	113.0	6.37
3	148.2	-
5	124.9	-
6	117.6	6.46
9	16.6	2.03

S8. ^{1}H and ^{13}C NMR chemical shifts of *3,4-dihydroxyphenethyl glycol*

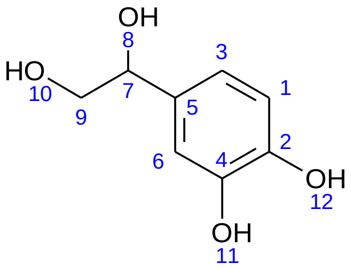

$C_8H_{10}O_4$	170.2 g/mol	DMSO-*d6*
Atom number	^{13}C (ppm)	^{1}H (ppm)
1	115.3	6.63
2	145.1	-
3	117.4	6.54
4	144.4	-
5	134.7	-
6	114.1	6.72
7	74.1	4.34
9	68.1	3.33

S9. ^{1}H and ^{13}C NMR chemical shifts of *N-[2-(3,4-dihydroxyphenyl)ethyl]-acetamide* (N-acetyldopamine)

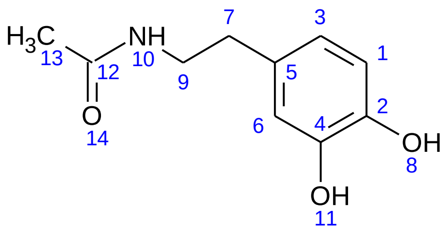

$C_{10}H_{13}NO_3$	195.2 g/mol	DMSO-*d6*
Atom number	^{13}C (ppm)	^{1}H (ppm)
1	115.3	6.61
2	143.9	-
3	119.6	6.42
4	145.4	-
5	130.7	-
6	116.4	6.56
7	35.2	2.49
9	41.0	3.14
12	169.4	-
13	23.1	1.77

S10. ^{1}H and ^{13}C NMR chemical shifts of *niacinamide*

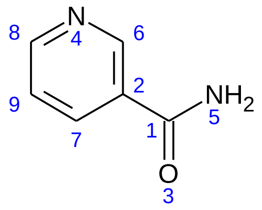

$C_6H_6N_2O$	122.1 g/mol	DMSO-*d6*
Atom number	^{13}C (ppm)	^{1}H (ppm)
1	166.9	-
2	130.1	-
6	149.0	9.03
7	135.5	8.20
8	152.3	8.70
9	123.9	7.50

S11. ^{1}H and ^{13}C NMR chemical shifts of *succinic acid*

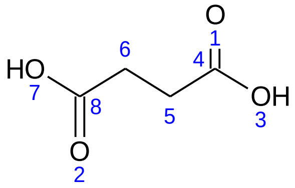

$C_4H_6O_4$	118.1 g/mol	DMSO-*d6*
Atom number	^{13}C (ppm)	^{1}H (ppm)
1	174.4	-
2	29.9	2.39
3	29.9	2.39
4	174.4	-
